# Heredity

**Published:** 1918-01

**Authors:** P. A. Zaring

**Affiliations:** Brownstown, Indiana


### HEREDITY.

**By P. A. Zaring, M.D., Brownstown, Indiana.**

The study of engenics has brought forward more definitely than it had been, the fact that a man or woman of intellect is not a mere accident, a freak of nature, but a product of evolution on regular lines. There is no such thing as chance generation in this process. An infinite number of causes has produced every man and woman just so. Some have great capacities for thinking; others have great capacities for doing; others with indifferent capacities are favored by circumstances so they succeed. Influences may be such that an ordinary person may make an extraordinary effort in some direction, and develop some latent power. He marries a wife that is similarly developed, and their children inherit something of these developments, a part of which is necessarily a propensity to further development on the same or similar lines. If favorable environments conspire with such inherent capacity and inclination, the individual will exceed others, perhaps to the extent termed genius. But it requires capacity, inclination, perseverance, and withal an opportunity. If circumstances had kept Thomas Edison in the cornfield all his life, we would never have heard of him, notwithstanding his capacity and perseverance. It is sometimes said that, given the capacity and the propensity, the individual will create his own opportunities; and sometimes it seems so. But where it seems so, some slight change of the combinations of conditions might have turned the fellow into an entirely different course where his whole life's career would have been different and the results according. Reduced to the simplest formbula we have Selection, Environment, and Use.

In the matter of wild plants, wild animals, and man, the selection is said to be natural. In the matter of domestic plants and animals, man does the selecting. Perhaps this is natural selection only a little more intensive. Man is as natural as any other animal. It is natural for him to do things by reason as for other animals to do things by instinct.

Sometimes, perhaps always, a line of development might be traced through a number of generations from the ordinary up to the master mind. It is not always possible to do so, becaus it is not always known who and what the ancestors were. But the law is a law just the same, and needs no lengthy discussion. Then it is perfectly plain that in any case the more perfectly developed the parents are the better is the chance of the offspring. Some apparent exceptions to this may be more apparent than real. Well developed parents, might, at a given age, produce bright children, and afterward become dissipated, and then produce inferior children. And even without seeming to be dissipated, the change (even slight) of environment, of mental bent, the difference of prospects, the condition of health, may tell on the offspring without being easy to account for.

But other things being equal, mature parents produce better children, physically, mentally, and morally, than parents who are premature. No certain age can be set down as indicating maturity. Some persons mature younger than others do, according to their zeal for improvement and their opportunities. But it were very exceptional indeed if any one should mature physically, mentally and morally, before the age of twenty-five, while many have not yet reached their best at forty. Of course they ought not, on the other hand, to produce children while in the decline of life. A man who is so doted as to rank with the feeble-minded would be expected to produce no better chilren than other feeble-minded persons. If nature were a good designer it would have rendered senile men sterile as well as women. It is so with extremely old men, as with all extreme neurotics. But in all these cases the line it not drawn close enough. We should assist nature by enforcing a more exteinsive prohibition.

When a man and woman are mature, and marry, the question remains, "What further can be done to render the prospective children as eligible as possible?"

It is the part of the father to be in the most perfect physical, mental, and moral condition at the time of procreation, that the children may be as eligible as possible. This is all he can do directly. He may further contribute indirectly to this end by helping to keep his wife in the proper condition, for all through gestation she has much to do, perhaps (as some have maintained) all to do, in fixing the inclinations of the child. This influence continues in some measure during nursing. This inherited condition, acted on by environments during life, determines the charact^er of the individual.

The capacity to do things is inherited from the father as much as from the mother. Some say more. It is more perhaps only when the father has the more capacity. Where both parents are equal the heritage should be equal. The capacity means much; but the inclination, the propensity ,the determination, the tenacity of purpose; the patience, industry, and perseverance, which will not relent, mean a great deal more. A child of good capacity may lack energy to accomplish anything worth while. Another lacks the tenacity of purpose; another is too visionary to be practical; another is too prodigal; another lacks conceit; another has too much conceit. So an ordinary mind, well poised, may accomplish more than the greater mind if deficient in purpose and fortitude.

It is not necessary, and would be wrong, to indulge, and pet, and spoil, a woman during gestation. That would render her petulant and whimsical, and would impress on the child an exacting disposition; and it would be apt to grow up thinking the world owed it a living. If the woman does not work, or if she is overworked, the child is apt to be averse to toil. She should take pride in her work, and do everything well. Her food should be wholesome and sufficient in quantity and variety; but if she indulges a capricious appetite, the child will be an epicure. Her environments should be clean, comfortable, and convenient; and her clothing should be comfortable and respectable; but if she is fastidious of dress and furnishings, the child will be fixed so for life. She should resign herself to the most perfect contentment with what she has. She should read much, and what is good. Light novels and daily gossip should be discarded by all means. She should read only such matter as she would wish the child to delight in during life. She should actually study what she reads, mastering the thoughts and sentiments represented, that the child may be an industrious and profound student. Her reading may be all on one line, or it may be varied, according as she can be the most profoundly interested. If she studies very exhaustively the best poetry during gestation, her child will be a poet, good or indifferent, according to the capacity inherited from the parents. If she is profoundly interested in science all the while, she will bring up a scientist. Or she can make of it a historian, mathematician, musician, what she will, by this intensive interest and study during gestation. "As the twig is bent, so is the tree inclined," does not mean to bend the twig down and let it fly back again at once; but to bend it and keep it bent. The plastic, nervous system of the child is bent, and kept bent, during gestation, exactly according to the bent of the mother's mind. If she is frivolous, whimsical, impatient, changeable, ill-tempered, despondent, unreliable, untruthful, dishonest, hypocritical, the child will inhert the same weaknesses. The father has no such power over the child—not for a minute.

Everything in our nature is inherited. But usage has given different names to different aspects of our mental and moral constitution. We inherit from our prehuman ancestors certain acquirements (education) of theirs, which we denominate animal instincts. Such are the love of life; the means for its maintenance, as the instinct of the infant to nurse, and defense of danger; the perpetuation of the species. We inherit from our savage human ancestors certain acquirements( education) of theirs, which we denominate human instincts. Such are bipedal locomotion; using our hands for prehension; and communicating our thoughst by means of language, words, signs, and characters. We inherit from our racial ancestors color, peculiar facial features, and certain propensities and pasisons in greater or less degree. We inherit something of nationality which marks us as German, French, Irish or other. Different races, and even different nationalities, develop peculiar capicities for thinking and doing, according as general condition have influenced them alike for a long while. We inherit family peculiarities from our immediate parents. This may not always appear. The resemblance may be more that of the grandparents, according to the Mendelian law. But it is a family favor all the same. Our instincts, mental capacities, and inclination, are inherited education. The particular language we learn, and all that we learn subsequently, is our education, more specifically considered. But the education we acquire depends on the capacity, inclination as, perhaps more than, the opportunities with which we are environed during life.

The sex of the child, as well as the physical and mental favor, is determined by prepotency. This means the dominating sexual energy. This depends upon age, physical health, inherited propensities, moral scruples, education, and with women, the time of the month. The husband may be prepotent at a given time determines the sex and resemblance of that particular child. On account of the alternating of prepotency, there are both girls and boys in the same family. Some men are always prepotent to their wives, and all their children are boys, and resemble their father. Some women are always prepotent to their husbands, and their children are girls, and resemble their mothers. But there are exceptions to this rule which baffle explanation, where the girls resemble the father and the boys the mother. Theories have been offered, but it would be too tedious to trace them out here.

But the inclinations of the child, whether it is to be patient or irritable, industrious or lazy, studious or playful, frivolous or grave, and even the choice of studies, music, drawing, history, oratory ,science—are determined by the mental condition of the mother during gestation. The spendthrift, the fop, the kleptomaniac, and all other such unhappy heritages, must be charged to the mothers, while giving them credit for endowing other children with honesty, industry, economy, and all similar virtues.

In certain regions where it has been very difficult to support families, it has been the practice of the natives to kill the surplus of babies. The weaklings were usually the first victims; and if there must be victims, this was right. As between the sexes the girls were more apt to be disposed of, the boys being saved for warriors. This produced an excess of boys. This led to polyandry. This excess of husbands produced sexual surfeit in women, and consequent prepotency of the men. The result was that an excess of male children was born, and polyarndry came to seem a necessity. In other countries, better favored by nature, the opulent have practical poygamy. In this case it was the men that surfeited, the women were prepotent, and an excess of female children succeeded. The same results may be observed in monogramous countries, in the families of lascivious persons who are not restrained by the rules and laws of marriage.

The child of a drunken, epileptic, or lunatic father is apt to be idiotic—fairly certain to be neurotic. But just what part of such a heritage comes directly from the father, and what part comes through the mother, because of the impressions made on her mind by the husband, would be variable and indeterminate. Of course when the heritage is from the mother, the ruin of the child is more certain and more easily understood. We all know that such parents should be prohibited from reproducing. But for that matter we know lots of things we cannot enforce.

The superiority of the younger members of large families of children has been too greatly recognized to require any lengthy discussion. When parents are of mature age and condition they produce better children than while they are premature. The first children help to mature the parents, fitting them to produce better children later. But this is at the cost of such first children. This should not be done in consequence of early marriage, unless even these first children are apt to be better than the average. Even if the couple are to produce a large family of children, with the chance of the younger members being better, still it is not best, as a rule, for them to marry young. Because the children produced before the parents were fully matured, would lower the average of that family, at least, if not of the community.

This is improved in the breeding of animals, and in the study of the human animal. Redfield and others have studied the master minds of the world, and find that every one of them had a good birth rank. The birth rank is the difference between the ages of the son and the father. The average birth rank for a given number of generations is determined by adding the birth rank and dividing by the given number. I was forty-six years old when my little son was born. My father was twenty-eight when I was born. His father was fifty-six when he was born, $56+28+46=130$; $130\div3=43\frac{1}{3}$, the boy's average birth rank for three generations. Another boy I know of has a living great grandfather who was fifty-one when he was born. $51\div3=17$, his average birth rank for three generations. Other things being equal, this would give my boy a great advatage of him in the journey to the goal of life. Now, since no great man has ever lived who did not have a good average birth rank for a number of generations, it is not likely that any one ever will or can, and that without any regard to and in spite of the worth of his ancestors. If the greatest men and women in the world should, while in their teens, produce children, and these children while in their teens should produce other children, and so continue for several generations, they would degenerate into an inferior race, in spite of environments.

Suppose the human could reproduce as young as many other animals; and suppose little children should reproduce before they learn to talk; and suppose this product should reproduce in turn before they learn to talk; and so on for a number of generation; we would expect that in a few generation the result would be a race in which language were impossible.

So with other human qualities: Self-consciousness appears in the average child at the age of three. In some it appears later in life. In a smaller some it does not appear at all. But whether it appears sooner or later, imagine a number of successive generations to be reproduced before the dawn of self-consciousness; the result would be a race without self-consciousness. There are other faculties which appear still later in life. Then it seems plain enough, certain enough, that when men and women marry before the higher faculties are fully developed, as reason, judgment, and imagination, degeneration of mental and moral worth are certain.

In the absence of statistics (and perhaps satisfactory statistics on the subject were impossible), I resorted to interviewing a number of teachers to ascertain what per cent. of pupils may be considered actually bright. The conclusion is about 5 per cent. Perhaps twice as many are idiots and dullards who do not learn anything. This leaves a middle class of about eighty-five per cent. called mediocre. This eighty-five per cent. might be subdivided into a large number of classes according to their inclinations and fitness for things. Then can work in the fields, quarries, mines, factories; they can learn trades, do business, scheme, cheat, and dissemble, some of them squeeze into the professions, but they cannot be truly professional, only business-like; they fill the most of the offices, and are very successful grafters; they make good soldiers, and sailors, and do very well in the civil service. But whatever requires profound thought, exhaustive reasoning, comprehensive judgment, or exalted flights of imagination, would be beyond them.

It is not to be understood that the five per cent. are all master minds. But all of the master minds of the world would be comprehended within this five per cent. All of the men and women of the world who materially advance civilization are surely within this fraction. Without this five per cent. there would be no great discoverers; no great inventors; no great orators; no good books; no civilization. There would be leaders of the people, to be sure. The lowest savages have leaders; and they suppose their chiefs and medicine-men are very important fellows. But even among savages it is the occasional genius, who is naturally superior to the others that saves the tribe from being worse than it is. Without the superior beings, savage tribes would never develop into civilized nations. And without this five per cent. of bright men and women our civilization could advance no further, and could not maintain at its present status.

The eighty-five per cent. can learn to do things according to established rules; but without the five per cent. there would be no rules. If the five per cent. of intellectuals were suddenly struck out of existence, the eighty-five per cent. would continue for a while to carry on the industries on established lines; but instead of a continuous amelioration of condition it would be a constant deterioration. They might be partly saved by a chance genius here and there; but even a chance genius originating outside of the five per cent. of intellectuals would be very rare indeed.

If this five per cent. of the most intellectual children were removed from the world in infancy, the second generation would have less, far less, than five per cent. of bright children. Remove these and the third generation would have still less, and so on down the ages.

No doubt ancient Athens produced the largest number of great intellects of any community of similar population in such a time as it was to the front of civilization. And yet all of those intellectuals were far within one per cent. of the population.

This five per cent. of intellectuals in this country, are practicing race suicide more than the others. Instead of studying the laws of heredity, and improving the race by obedience to these laws, the tendency is toward worse conditions.

